# From Liver Cirrhosis to Cancer: The Role of Micro-RNAs in Hepatocarcinogenesis

**DOI:** 10.3390/ijms22031492

**Published:** 2021-02-02

**Authors:** Raphael Mohr, Burcin Özdirik, Joeri Lambrecht, Münevver Demir, Johannes Eschrich, Lukas Geisler, Teresa Hellberg, Sven H. Loosen, Tom Luedde, Frank Tacke, Linda Hammerich, Christoph Roderburg

**Affiliations:** 1 Department of Hepatology and Gastroenterology, Campus Virchow Klinikum (CVK) and Campus Charité Mitte (CCM), Charité University Medicine Berlin, Augustenburger Platz 1, 13353 Berlin, Germany; burcin.oezdirik@charite.de (B.Ö.); joeri.lambrecht@charite.de (J.L.); muenevver.demir@charite.de (M.D.); johannes.eschrich@charite.de (J.E.); lukas.geisler@charite.de (L.G.); teresa.hellberg@charite.de (T.H.); frank.tacke@charite.de (F.T.); Linda.Hammerich@charite.de (L.H.); christoph.roderburg@charite.de (C.R.); 2 Clinic for Gastroenterology, Hepatology and Infectious Diseases, Medical Faculty of Heinrich Heine University Düsseldorf, University Hospital Düsseldorf, Moorenstraße 5, 40225 Düsseldorf, Germany; Sven.Loosen@med.uni-duesseldorf.de (S.H.L.); tom.luedde@med.uni-duesseldorf.de (T.L.)

**Keywords:** miRNA, hepatocellular carcinoma, biomarker, translational

## Abstract

In almost all cases, hepatocellular carcinoma (HCC) develops as the endpoint of a sequence that starts with chronic liver injury, progresses to liver cirrhosis, and finally, over years and decades, results in liver cancer. Recently, the role of non-coding RNA such as microRNA (miRNA) has been demonstrated in the context of chronic liver diseases and HCC. Moreover, data from a phase II trial suggested a potential role of microRNAs as therapeutics in hepatitis-C-virus infection, representing a significant risk factor for development of liver cirrhosis and HCC. Despite progress in the clinical management of chronic liver diseases, pharmacological treatment options for patients with liver cirrhosis and/or advanced HCC are still limited. With their potential to regulate whole networks of genes, miRNA might be used as novel therapeutics in these patients but could also serve as biomarkers for improved patient stratification. In this review, we discuss available data on the role of miRNA in the transition from liver cirrhosis to HCC. We highlight opportunities for clinical translation and discuss open issues applicable to future developments.

## 1. Introduction

The incidence of hepatocellular carcinoma (HCC) has been steadily increasing over the last decades. It was only most recently that a reversal of this trend was observed in Western countries [1]. Nevertheless, HCC ranks number five of the most common cancers worldwide and is one of the leading causes of cancer-related deaths, presenting a major global health problem [2,3]. The majority of HCC develops in the context of chronic liver inflammation and cirrhotic transformation, e.g., due to viral hepatitis, alcohol-related liver damage, and nonalcoholic fatty liver disease [4].

The degree of liver injury and the tumor stage jointly determine the prognosis of patients with HCC, which often remains poor. In early-stage disease, surgery is the curative treatment of choice. Patients with limited tumor burden may also be considered for liver transplantation [5]. However, many patients are diagnosed with advanced tumor stages and left to palliative treatments only. In these patients, pharmacological treatment options for systemic therapy have greatly improved over the past years, but their efficacy is still not satisfying. Thus, there is an unmet need for novel treatment options to further improve patients’ prognosis.

In this context, microRNAs (miRNAs) represent an important tool to gain new insights into the molecular pathogenesis of HCC. In this review, we summarize available data on the role of miRNA in hepatocarcinogenesis. We will briefly recapitulate the current algorithms for systemic treatment and discuss the role of miRNA in tumor biology and whether they could serve as therapeutic targets for disease modulation and predictors of treatment response.

## 2. Current and Emerging Therapeutic Options for HCC

Continuous viral (e.g., chronic hepatitis B, C, delta co-infection), toxic, or metabolic liver injury leads to chronic liver inflammation and conditions the transformation towards fibrosis and cirrhosis. This slow transition sets the basis for hepatocarcinogenesis and HCC progression. Despite the recommendation of surveillance and periodic imaging of cirrhotic livers, many patients are diagnosed with intermediate or advanced stages of HCC according to the Barcelona Clinic of Liver Cancer (BCLC) staging system [6]. Pharmacological treatment of HCC is particularly challenging as HCCs show important tumor heterogeneity and arise from a distinct microenvironment, with regard to different etiologies of liver injury, and different degrees of inflammation and fibrotic/cirrhotic transition.

Until recently, systemic treatment options of advanced HCC were limited to tyrosine-kinase inhibitors (TKI) [7]. In 2008, the SHARP trial established sorafenib, which simultaneously inhibits tumor growth by targeting the Raf-MEK-ERK cascade as well as angiogenesis by targeting vascular endothelial growth factor (VEGFR) 2, platelet-derived growth factor receptors (PDGFR), and KIT as a novel treatment in patients with advanced HCC [8]. Sorafenib remained the only standard systemic treatment for HCC for almost one decade. In 2018, lenvatinib, a molecule targeting VEGFR 1–3, fibroblast growth factor receptor (FGFR) 1–4, PDGFR, RET, and KIT [9], was tested as non-inferior in the REFLECT trial. Based on data from the RESORCE and CELESTIAL studies, both regorafenib and cabozantinib, targeting VEGFR 1–3, as well as the MET and AXL pathway [10], are approved for use in patients refractory to sorafenib [11,12]. Nevertheless, high toxicity rates and moderate effectivity limit the use of TKIs. Ramucirumab, a novel antibody directed against VEGFR 2, has demonstrated efficacy when used in patients with elevated serum alpha-fetoprotein (AFP) levels [13].

Immunotherapies seemed particularly promising in the setting of HCC, since cirrhosis bears an immunosuppressive environment that may be modulated by checkpoint inhibitors [14]. During the induction of an immune response, tumor-associated antigens are presented by antigen-presenting cells to T-cells, which become activated and induce tumor cell death. This process is negatively regulated by immune checkpoints, such as Programmed Cell Death 1 Protein (PD-1), a receptor mainly expressed on activated lymphocytes. Binding of PD-1 by its ligands PD-L1 and PD-L2 inhibits T cell activation and results in immunosuppression. Therefore, preventing activation of the PD-1/PD-L1 pathway might restore the ability of immune cells to recognize and kill tumor cells. Indeed, phase I/II data suggested that PD-1/PD-L1 inhibitors could be an effective anti-HCC tool, but phase III studies showed limited efficacy when applied as single agents [15]. However, when used as combination therapy including different substance classes, the combination of atezolizumab (PD-L1 antibody) plus bevacizumab (VEGF antibody) showed significantly improved overall survival (OS), progression-free survival (PFS), and excellent tumor response in the phase III IMBRAVE-150 study [16,17]. Current guidelines incorporate this evidence and both sorafenib and lenvatinib might be moved to subsequent therapy lines after immunotherapy failure. Other combinations are currently tested and might lead to further changes of treatment algorithms in the near future. Further progress in immunotherapy for HCC will critically rely on the identification of predictive biomarkers that allow early identification of ‘responders’ in order to personalize treatments as early as possible [18]. 

## 3. Role of miRNAs

MicroRNAs (miRNAs) represent a class of small, single-stranded RNAs of approximately 22 nucleotides length that were first described in *C. elegans* by the group of Ambros [19,20,21,22,23]. MiRNAs do not encode for proteins but repress the expression of their target RNA both on the transcriptional and translational level. 

**Figure 1:**
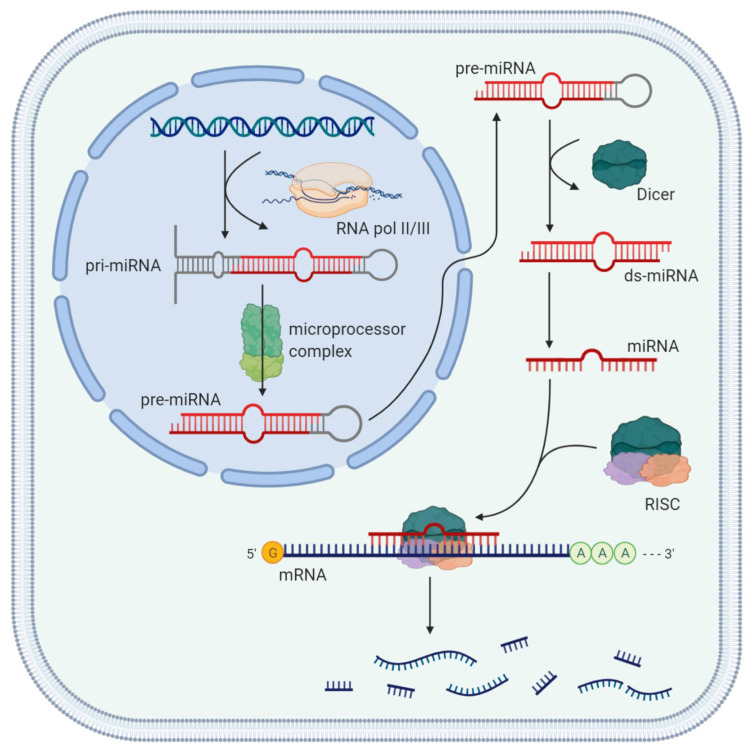
Cycle of microRNAs. Abbreviations: microRNA (miRNA), RNA-induced silencing complex (RISC), double-stranded miRNA (ds-mi RNA), messenger RNA (mRNA). Created with Biorender.com.

MiRNAs are transcribed by RNA polymerase II and III, leading to 500–3000 nucleotides long pri-miRNAs that are processed in the nucleus by the so-called “microprocessor complex” into precursor miRNAs (pre-miRNA, approximately 70 nucleotides long). Pre-miRNAs reach the cytoplasm via an exportin-5-mediated nuclear export. They are cleaved by the RNase III endonuclease “Dicer” into ~22 nucleotides long, double-stranded miRNAs. The single-stranded, mature miRNA is bound by Argonaute and integrated into the RNA-induced silencing complex (RISC). This complex is able to repress gene expression post-transcriptionally or translationally via binding of the loaded miRNA to the 3′ or 5′ UTR of its target messenger RNAs (mRNAs) (Figure 1). In case of complete complementarity, degradation of the target mRNA occurs, while in case of partial complementarity, translational repression is observed [19,21,24,25].

Up to now, more than 1800 miRNAs have been identified in humans [26]. In silico data predicted that more than 45,000 miRNA target sites are present in human DNA and that expression of more than 60% of all protein-coding genes are regulated by miRNAs [27]. Since one miRNA is able to influence expression of a whole networks of genes, many miRNAs are involved in the regulation of essential cellular processes and were associated with different disease states, such as acute and chronic liver diseases including viral hepatitis, steatohepatitis, liver fibrosis, cirrhosis, and HCC [28,29]. Since miRNAs are extremely stable in body fluids such as serum samples, they have been extensively studied in recent years in order to explore their potential as biomarkers for liver diseases [30].

## 4. Principal Physiological and Pathogenic Mechanisms of miRNAs 

One of the most studied miRNAs is miRNA-122, which accounts for approximately 70% of all miRNAs found in hepatic tissue. When chronic liver injury occurs, decreasing levels of miRNA-122 are observed, leading to the subsequent upregulation of multiple pro-fibrogenic factors such as Kruppel-like factor 6 (KLF6) [31,32]. Downregulation of miRNA-122 also affects a large network of genes involved in systemic iron homeostasis (via upregulation of bone morphogenetic protein receptor type 1A (Bmpr1a), hemochromatosis (Hfe), hemojuvelin (Hjv), and hepcidin antimicrobial peptide (Hamp) [33]), in lipid metabolism [34], cell differentiation [35], and circadian regulation [36]. Other miRNAs playing key roles in hepatocyte proliferation and liver regeneration are miRNA-24 and miRNA-34a [37]. Both negatively regulate hepatocyte nuclear factor 4 alpha (HNF4α) expression in vitro, resulting in the suppression of cytochrome P450 and a reduced amount of HepG2 cells in S-phase [37]. After partial hepatectomy, levels of miRNA-26a and miRNA-217 are decreased in hepatic tissue, stimulating hepatocyte proliferation through regulation of cyclin-D2 (CCND2) and cyclin-E2 (CCNE2) protein expression, B-cell lymphoma protein homolog (Bcl6), and N-lysine methyltransferase SETD8 [38,39]. As major regulators of gene expression, miRNAs also are involved in liver development [40]. The conditional knockout of DICER1, a key element of miRNA biogenesis, led to a significant decrease of miRNA-194, miRNA-192, and miRNA-122 in hepatoblast-derived liver cells. However, while mice with *AfpCre;Dicer1^flox/flox^* genotype, i.e., conditional deletion of DICER1 in liver parenchymal cells, displayed no phenotypic anomalies just after birth, at 2–4 months of age, they showed various signs of progressive liver damage, including increased cellular proliferation and apoptosis, elevated circulating alanine aminotransferase (ALT) and aspartate aminotransferase (AST) levels, and overall increased liver mass [41]. This highlights the importance of miRNAs in maintaining liver homeostasis, suggesting a key role in the progression of liver disease.

## 5. Clinical Application of miRNAs

Increasing insight into the mechanisms of miRNAs in liver disease make them an attractive tool and target for therapeutic approaches. Indeed, several studies have investigated the potential effect of miRNA (ant)agonists for dampening liver disease progression. Scarce data are available regarding the role of miRNAs in hepatocarcinogenesis. Since decreased expression of miRNA-26a in HCC tissue facilitates the rapid proliferation of hepatocytes [38], increasing miRNA-26a levels might be used as a therapeutic approach in HCC. Indeed, systemic administration of miRNA-26a using adeno-associated virus (AAV) vectors in an HCC mouse model resulted in significant inhibition of cancer cell proliferation, induction of tumor-specific apoptosis, and an overall protection from disease progression [42]. MiRNA-122, which is downregulated in HCC tissue and targets multiple pathways of HCC pathogenesis, has been proposed as a therapeutic target as well. LNP-DP1, a cationic lipid nanoparticle formulation, was used as a vehicle for miRNA-122 delivery into HCC cells. In vivo intra-tumoral injection resulted in a 50% suppression of HCC growth in xenografts within 30 days, which correlated well with suppression of target genes and impairment of angiogenesis [43]. The expression of various ATP-binding cassette (ABC) transporters, responsible for chemotherapy resistance, is regulated through miRNAs [44]. Therefore, miRNA modulation may also bear a potential to overcome mechanisms of chemotherapy resistance. Miravirsen, an antisense of miRNA-122, that prevents binding to viral RNA and therefore compromising HCV replication, was investigated as a therapeutic approach in viral hepatitis [45]. The potential to reduce hepatitis C RNA levels in a dose-dependent manner was demonstrated in chronic HCV-infected chimpanzees [46] and subsequently in clinical trials [47]. The application of MRX34, a liposomal miRNA-34a mimic, was evaluated as therapy of solid tumors, including HCC, but clinical trials were suspended due to significant immune-related adverse effects [48].

## 6. Specific miRNAs Involved in Hepatocarcinogenesis

**Figure 2:**
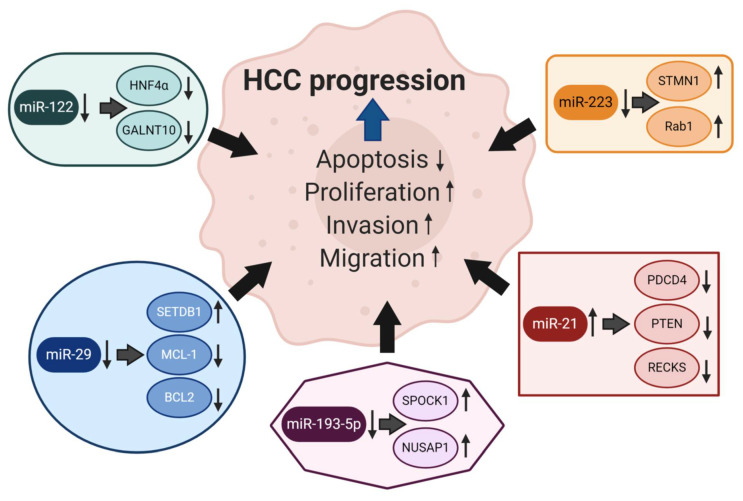
Overview of mechanisms of important miRNAs (expressed in liver tissue) in hepatocarcinogenesis. Created with Biorender.com.

A general overview of specific miRNAs and signaling pathways that are involved in hepatocarcinogenesis is provided in Table 1 and Figure 2.

### 6.1. miRNA-223

X-chromosome linked miRNA-223 is considered a neutrophil-specific miRNA since it is highly expressed in these cells, playing a pivotal role in attenuation of neutrophil maturation and activation [101]. It is one of the key regulators in homeostasis of the immune system (hematopoietic differentiation), in systemic inflammatory processes, and in various liver diseases [102,103]. MiRNA-223 modulates hepatocellular function by affecting cholesterol levels, drug metabolism, apoptosis, and chromosomal stability of hepatocytes.

In HCC, miRNA-223 is downregulated by sulfatide in association with reduced recruitment of acetylated histone H3 and C/EBPα to the pre-miRNA-223 gene promoter [49]. Wong et al. demonstrated a simultaneous overexpression of the downstream target Stathmin 1 (STMN1), a microtubule-regulatory protein, controlling cellular proliferation and S-phase of the cell cycle [50]. Dong et al. described the miRNA-223-dependent modulation of the mechanistic target of rapamycin (mTOR) signaling pathway by inhibiting cell growth and inducing apoptosis through Ras-related protein 1 (Rab1) [53]. Interestingly, overexpression of miRNA-223 inhibits the development of metastasis by targeting integrin αV [49]. As levels of miRNA-223 are decreased in serum of HCC patients, it might serve as a biomarker [104,105], especially as a monitoring tool in the context of systemic HCC treatment or liver transplantation [106,107].

### 6.2. miRNA-21

Located on chromosome 17q23.2, miRNA-21 is one of the most abundant miRNAs detected in the circulation and is widely expressed in various types of human tissues (bone marrow, liver, lung, kidney, intestine, colon, and thyroid) [58,108]. On a cellular level, it is located in the cytosol and extracellular exosomes [109,110]. miRNA-21 plays a significant role in inflammation, fibrosis, and especially carcinogenesis. It is overexpressed in multiple solid tumors (e.g., breast, colon, lung, pancreas, prostate, stomach, gall bladder, liver) [55,111]. 

In HCC, miRNA-21 is significantly upregulated in both tissue and serum [51,56,57,112]. An aberrant expression of miRNA-21 may contribute to HCC progression by modulation of phosphatase and tensing homolog (PTEN) and PTEN-dependent pathways, leading to increased cell invasion, migration, and proliferation. More specifically, upregulation of miRNA-21 decreases PTEN expression, causing increased activity of AKT and the mTOR kinase pathways. As a result, downstream mediators of PTEN such as tyrosine phosphorylation of focal adhesion kinase (FAK) and the expression of matrix metallopeptidase (MMP) 2 and 9 are modulated. Liu et al. described simultaneous silencing in Programmed Cell Death 4 (PDCD4) and reversion-inducing cysteine-rich protein with Kazal motifs (RECKS), leading to reduced apoptosis and increased cell invasion [54]. Exosomal miRNAs such as miRNA-21 are involved in intercellular communication, tumor microenvironment, and tumor metastasis [113]. Several studies revealed a link between increased serum levels of miRNA-21 and tumor progression [51,57,58,114,115]. Tomimaru et al. found miRNA-21 to be a more specific biomarker compared to AFP, when differentiating HCC from chronic hepatitis or healthy controls [57]. Zhou et al. established a plasma miRNA panel containing seven miRNAs, including miRNA-21, which provides high accuracy in the diagnosis of early-stage hepatitis B-related HCC [51]. 

### 6.3. miRNA-193a

miRNA-193a is a member of the miRNA-193 family and is located on chromosome 17q11.2 [60]. Pre-miRNA-193a generates two mature miRNAs, miRNA-193a-3p and miRNA-193a-5p, which differ in distinct target sets for each miRNA [116]. Both act as tumor suppressors in liquid and solid malignancies [63,65,66,116,117,118], whereas irregular miRNA-193a expression significantly promotes carcinogenic conditions [117]. When expressed at physiological levels, miRNA-193a-3p mediates tumor-suppressive effects through Epidermal Growth Factor Receptor (EGFR) signaling, enhances apoptosis by inhibition of MCL1, and suppresses tumor cell migration and invasion through small GTPase Rab27B or Erb-B2 Receptor Tyrosine Kinase 4 (ERBB4) and S6K2 [63,64,65,66]. The miRNA-193a gene is frequently deleted in several types of human tumors and loss of miRNA-193a-3p’s anti-tumor functions may contribute to neoplastic transformation [64]. 

In the context of HCC, the expression of miRNA-193a-5p in tumor tissue is controversially discussed as different observations have been reported. In line with most published studies, Roy et al. identified downregulation of miRNA-193a-5p as a common feature of murine and human HCC regardless of the underlying etiology [59]. Downregulation of miRNA-193-a-5p causes cell proliferation and inhibits apoptosis via overexpression of Nucleolar and Spindle-Associated Protein 1 (NUSAP1) and cysteine-rich acidic secreted protein/osteonectin, cwcv, and kazal-like domains proteoglycan 1 (SPOCK1), a common target gene of miRNA-139-5p, miRNA-940, and miRNA-193a-5p [59,62]. Conversely, Wang et al. described an overexpression of miRNA-193a-5p in HCC, targeting Bcl2-Modifying Factor (BMF), which modulates cell proliferation, G1/S transition, and apoptosis [60].

Loosen et al. identified miRNA-193a-5p as a potential biomarker in the context of HCC as circulating relative miRNA-193a-5p levels were significantly elevated and predictive for patients’ outcome after tumor resection [61]. In line with this, Liu et al. described a significant difference in miRNA-193-a-5p levels in serum of HCC patients compared to non-HCC patients, without any difference among patients with liver cirrhosis, chronic hepatitis B, and healthy controls [119]. Hydbring at al. demonstrated that targeting miRNA-193a-3p causes cell cycle arrest and apoptosis of cancer cells in different tumor types, such as triple-negative breast cancers and gastric cancers [64]. In the context of HCC, Salvi et al. transfected HCC cells with miRNA-193a, causing increased apoptosis and decreased proliferation. In combination with sorafenib, further inhibition of HCC proliferation could be observed [120].

### 6.4. miRNA-122

miRNA-122, located on chromosome 18, is the most abundant miRNA in the liver and plays a central role in a large variety of biological processes such as homeostasis, metabolism (regulation of fatty acid metabolism and cholesterol), and liver development (hepatocyte proliferation, differentiation, maturation, and polyploidy) [72,121,122,123]. Transcription of miRNA-122 is regulated by liver-enriched transcription factors, including CCAAT/enhancer-binding protein (C/EBP) α, hepatocyte nuclear factor (HNF) 1α, HNF3β, and HNF4α [72]. miRNA-122 is downregulated in HCC tissue, being associated with hepatocarcinogenesis, metastasis, and poor prognosis [28,67,68]. Accordingly, overexpression of miRNA-122 suppresses HCC cell proliferation and increases chemosensitivity of HCC to antitumoral agents [28,67,70]. Several signaling pathways are involved in miRNA-122-mediated tumor suppression, including cyclin G1, pyruvate kinase isoform M2 (PKM2), Wnt family member 1 (WNT1), and paternally expressed gene 10 (*PEG10*) [67,68,73,74]. Wu et al. described a correlation between reduced miRNA-122 expression in hepatitis B-related HCC and venous invasion as well as poor prognosis by inhibition of hepatocyte nuclear factor 4α (HNF4α) and UDP-N-acetyl-α-D-galactosamine polypeptide N-acetylglucosaminyltransferase-10 (*GALNT10*) [75]. 

miRNA-122 may be a useful biomarker for detecting early liver injury [124,125,126] as it is released in response to various inflammatory processes such as viral infections and hepatocellular malignancies [127]. Nine plasma miRNAs, including miRNA-122, have been identified as biomarkers that predict regorafenib response in patients with HCC [128]. However, recent data challenge the idea of miRNA-122 as a diagnostic biomarker by revealing large interindividual and intraindividual variability of miRNA-122 levels in serum among healthy volunteers [129]. 

In the context of miRNA-122-based targeted therapy, long non-coding RNA HOTAIR, an oncogene in multiple cancers, might play an important role since it negatively regulates miRNA-122 expression in HCC cells by DNA methyltransferase-mediated DNA methylation and Cyclin 1 activation. Cheng et al. demonstrated that knockdown of HOTAIR was sufficient to inhibit tumorigenicity in vitro and in vivo by upregulation of miRNA-122 expression [130]. 

A phase 2a, randomized, double-blind study investigated miravirsen, a miRNA-122 inhibitor, as treatment for chronic HCV infection (NCT01200420). Miravirsen showed prolonged dose-dependent reductions in HCV RNA levels without viral resistance in chronic hepatitis C patients [47,131]. Several clinical trials are currently ongoing, e.g., exploring miRNA-122′s role as a marker for detection of drug-induced liver injury following chemotherapy (NCT03039062), its prognostic and predictive value for clinical outcome in patients with acute liver failure (NCT03000621), and the effect of direct-acting antivirals on miRNA-122 and insulin resistance in chronic HCV patients (NCT0300062).

### 6.5. miRNA-29

The miRNA-29 family consists of miRNA-29a, miRNA-29b-1, miRNA-29b-2, and miRNA-29c and is located on chromosomes 7q32.3 and 1q32.2. miRNA-29 is a critical player in multiple processes, including fibrosis, angiogenesis, epigenetics, proteostasis, metabolism, proliferation, apoptosis, metastasis, and immunomodulation [132,133,134]. It’s role as a tumor suppressor and oncogene is discussed controversially [76,77,133]. miRNA-29a/b/c expression is downregulated in patients with advanced liver fibrosis and mice with fibrosis induced by carbon tetrachloride (CCl4) or bile duct ligation [135]. More specifically, transforming growth factor beta (TGF-β) and nuclear factor kappa B (NF-κB)-dependent downregulation of miRNA-29 promotes the expression of extracellular matrix genes, such as Col1a1, Col4a5, and Col5a3, in hepatic stellate cells [132]. Matsumoto et al. demonstrated improved liver fibrosis in CCl4- and thioacetamide (TAA)-induced fibrosis models after treatment with miRNA-29a, indicating its important role as a potential target and therapeutic tool in liver fibrosis [135]. Downregulation of miR-29 is observed in various types of cancers including HCC and is associated with poor survival [76,77,136]. Parpart et al. described AFP as a functional antagonist of miRNA-29, contributing to global epigenetic alterations and poor prognosis in HCC. AFP inhibits miRNA-29a/b-1 transcription through binding of c-Myc to its transcript [76]. miRNA-29 contributes to the modulation of several genes, such as the upregulation of SET domain bifurcated 1 (SETDB1), an H3K9-specific histone methyltransferase, which is significantly associated with HCC disease progression, cancer aggressiveness, and poorer prognosis [79]. MiRNA-29a/b/c promotes apoptosis of HCC cells by suppressing two cell survival genes, MCL-1 and BCL2 [77]. MiRNA-29 acts as a tumor suppressor miRNA in a Myc- and AKT/Ras-induced HCC mouse model [79,137]. Despite miRNA-29′s potential as a prognostic biomarker, only a few studies have investigated its significance in HCC patients [138]. 

### 6.6. miRNA34a/c

The miRNA-34 family consists of miRNA-34a, miRNA-34b, and miRNA-34c and is located on chromosomes 1 and 11. While miRNA-34a is encoded by its own transcript, miRNA-34b and miRNA-34c share a common primary transcript [139,140]. Acting as a tumor-suppressor, miRNA-34 members modulate the p53 pathway by targeting c-MYC, CDK6, and c-MET, and therefore affect proliferation, apoptosis, and invasion in many cancer types, including pancreas, prostate, brain, colon, and breast cancer [80,88,89,90,91]. In hepatic cells treated with ethanol, expression of miRNA-34a was demonstrated to promote proliferation, migration, and transformation by targeting caspase-2 and sirtuin 1, which are involved in tissue remodeling during disease progression from normal liver through cirrhosis to HCC [92]. 

In the context of HCC, miRNA-34 is downregulated [80,81,82,83,84,85,87]. In early stages of liver regeneration, miRNA-34a is negatively correlated to the expression of Notch receptors [87]. By using miRNA-34a mimics, Wang et al. demonstrated that the Notch signaling pathway led to inhibition of cell growth, cell cycle arrest in G2/M phase, and increased cell apoptosis rate [87]. MiRNA-34a regulates histone deacetylase 1 (HDAC1), which inhibits HCC cell proliferation and induces apoptosis [81]. Moreover, miRNA-34a negatively regulates the expression of lactate dehydrogenase A (LDHA), which inhibits LDHA-dependent glucose uptake in cancer cells, as well as cell proliferation and invasion [80]. 

miRNA-34c-3p, one of the mature miRNAs of miRNA-34c, directly targets myristoylated alanine-rich protein kinase c substrate (MARCKS), the most prominent cellular substrate for protein kinase C, binding calmodulin, actin, and synapsin. Song et al. demonstrated that knock-out of MARCKS in HepG2 cells reduces cell migration and invasion, but not cell proliferation [84]. Liu et al. showed that miRNA-34c-5p alleviates HCC progression by negatively regulating FAM83A level [85]. FAM83A acts as a cancer-metastasis promoter, which accelerates migration, invasion, and metastasis, by forming a FAM83A/PI3K/AKT/c-JUN positive-feedback loop to activate epithelial-to-mesenchymal transition (EMT) signaling [141]. More specifically, FAM83A activates the PI3K/AKT signaling pathway and its downstream target c-JUN protein, as well as EMT proteins such as E-cadherin (downregulated), Vimentin, and N-cadherin (upregulated) [141].

Several studies provided evidence that miRNA-34a-5p may serve as a potential biomarker for liver cirrhosis since it is elevated in the serum of cirrhotic patients with no further increase in HCC patients and significantly correlates with the expression of AST, a reliable marker for liver damage [119,142]. On the other hand, low expression of miRNA-34c predicts poor prognosis in HCC as it is linked to advanced tumor stage and metastatic disease [84]. A synthetic miRNA-34a mimic is currently investigated for treatment of patients with primary liver cancer and liver metastases (NCT01829971) [143]. miRNA-34a may have potential as a therapeutic tool in metastatic disease, chemoresistance, and tumor recurrence [140,143,144].

### 6.7. miRNA-199

The miRNA-199 family consists of miRNA-199a and miRNA-199b, located on chromosomes 19 and 1. miRNA-199 is the third most abundant miRNA in liver tissue and is of great interest for cancer therapies since several potential targets of miRNA-199 are involved in carcinogenesis and metastatic progression [100]. Irregular expression of miR199a/b has been observed in various types of cancer, e.g., skin, pancreas, lung, stomach, and lymphoma. In non-small cell lung cancer, for example, the upregulation of miRNA-199a/b inhibits cell proliferation, migration, and invasion by inhibition of Axl expression [95]. 

Upregulation of miRNA-199 plays a key role in progression of chronic liver injury to liver fibrosis and advanced cirrhosis [145]. Contrarily, miRNA-199 is downregulated in HCC compared to normal liver tissue [93] and linked to the regulation of mTOR, c-Met, hypoxia-inducible-factor 1 (HIF-1)α, and CD44 [96,97,98,99]. MiRNA-199a/b-5p acts as a HCC-specific tumor suppressor, which inhibits Rho-associated protein kinase 1 (ROCK1) and modulates ROCK1/MLC and PI3K/AKT pathways, which are essential for HCC progression [100]. Emerging evidence suggests a potential role of miRNA-199a as a serum biomarker to detect patients with HCC [94,146,147]. Regarding miRNA-199′s therapeutic potential, Callegari et al. developed a miRNA-199-dependent oncolytic adenovirus [148].

## 7. Conclusion and Perspectives

MiRNAs are involved in hepatocarcinogenesis. This fact makes them interesting biomarkers for reflecting HCC pathogenesis as well as putative targets for preventing or treating HCC. Both in vitro and in vivo data argue for a potential therapeutic use of small RNAs in liver cancer. The principal suitability of miRNAs as a target in liver diseases has been demonstrated for Miravirsen in the context of hepatitis-C virus infection. Nevertheless, several challenges are still to be overcome before RNA-based therapies in the setting of HCC can be translated into clinical routine. 

In this review, we summarized current knowledge on non-coding RNA in the transition from liver cirrhosis to HCC. We highlighted opportunities for clinical translation and discussed open issues applicable to future developments. 

## Figures and Tables

**Table 1:** Overview of important miRNAs in hepatocarcinogenesis. Abbreviations: Stathmin 1 (STMN1), Ras-related protein 1 (Rab1), phosphatase and tensing homolog (PTEN), Programmed Cell Death 4 (PDCD4), reversion-inducing cysteine-rich protein with Kazal motifs (RECKS), metalloproteinkinase inhibitor 3 (TIMP3), Nucleolar And Spindle-Associated Protein 1 (NUSAP1), secreted protein/osteonectin, cwcv, and kazal-like domains proteoglycan 1 (SPOCK1), Mcl-1 (myeloid cell leukemia 1), Erb-B2 Receptor Tyrosine Kinase 4 (ERBB4), Wnt family member 1 (WNT1), paternally expressed gene 10 (*PEG10*), pyruvate kinase isoform M2 (PKM2), hepatocyte nuclear factor 4α (HNF4α), UDP-N-acetyl-α-D-galactosamine polypeptide N-acetylglucosaminyltransferase-10 (*GALNT10*), KLF6 (Kruppel-like factor 6), Bcl-2 (B-cell lymphoma 2), SET domain bifurcated 1 (SETDB1), Notch homolog 1, translocation-associated (NOTCH1), Histone deacetylase 1 (HDAC1), myristoylated alanine-rich protein kinase c substrate (MARCKS), Cyclin-dependent Kinase 6 (CDK6), sirtuin 1 (SIRT1), mechanistic Target of Rapamycin (mTOR), hypoxia-inducible-factor 1 (HIF-1)α, Rho-associated protein kinase 1 (ROCK1).

miRNA	Expression in Liver Tissue	Level in Circulation	Functions in HCC	Selected Targets
**miR223**	↓ [49,50]	↓ ↑ [51,52]	Inhibition of cell growth, induction of apoptosis [53]	STMN [50], Rab1 [53], integrin αV [49]
**miR-21**	↑ [54,55]	↑ [52,56,57]	increased cell invasion, migration, proliferation	PTEN [54,55], PDCD4, RECKS [54], TIMP3 [58]
**miR-193**	↓ ↑ [59,60]	↑ [61]	Increased cell proliferation, inhibition of apoptosis [33]	NUSAP1 [33], SPOCK1 [59,62], MCL1, ERBB4, S6K2 [63,64,65,66]
**miR-122**	↓ [67,68]	↑ [28,52,69,70,71]	hepatocarcinogenesis, forming metastasis [28,67,68]	CUTL1 [72], WNT1, *PEG10*, PKM2 [67,68,73,74], HNF4α, *GALNT10* [75], KLF6 [31,32]
**miR-29**	↓ [76,77]	↑ [78]	promotes apoptosis [77], associated with HCC disease progression, cancer aggressiveness [79]	MCL-1, BCL2 [77], SETDB1 [79], DNMT3A [76]
**miR-34**	↓ [80,81,82,83,84,85]	↓ [86]	inhibition of cell growth, increase in cell apoptosis rate [87]	NOTCH1 [87], HDAC1 [81], MARCKS [84], FAM83A [85], c-MYC, CDK6, c-MET [80,88,89,90,91], caspase-2, SIRT1 [92], BCL2
**miR-199**	↓ [93]	↓ [94]	inhibits cell proliferation, migration and invasion [95]	mTOR, c-Met, HIF-1α, CD44 [96,97,98,99], ROCK1 [100], Axl [95]

## Abbreviations

AAV	adeno-associated virus
ALT	alanine aminotransferase
AFP	alpha-fetoprotein
AST	aspartate aminotransferase
ABC	ATP-binding cassette
BCLC	Barcelona Clinic of Liver Cancer
BMF	Bcl2-modifying factor
C/EBP	CCAAT/enhancer-binding protein
ds-mi RNA	Double-stranded miRNA
FAK	focal adhesion kinase
ERBB4	Erb-B2 Receptor Tyrosine Kinase 4
HCC	hepatocellular carcinoma
HNF	hepatocyte nuclear factor
HNF4α	hepatocyte nuclear factor 4 alpha
KLF6	Kruppel-like factor 6
MMP	matrix metallopeptidase
mTOR	mechanistic target of rapamycin
mRNA	messenger RNA
miRNA	microRNA
ncRNA	non-coding RNAs
NUSAP1	Nucleolar and Spindle-Associated Protein 1
OS	overall survival
PEG10	paternally expressed gene 10
PTEN	phosphatase and tensing homolog
PDGFR	platelet-derived growth factor receptors
PD-1	programmed cell death 1
PDCD4	programmed cell death 4
PFS	Progression-free survival
PKM2	pyruvate kinase isoform M2
Rab1	Ras-related protein 1
RECKS	reversion-inducing cysteine-rich protein with kazal motifs
RISC	RNA-induced silencing complex
SPOCK1	secreted protein/osteonectin, cwcv, and kazal-like domains proteoglycan 1
SETDB1	SET domain bifurcated 1
STMN1	Stathmin 1
TKI	tyrosine-kinase inhibitors
GALNT10	UDP-N-acetyl-α-D-galactosamine polypeptide N-acetylglucosaminyltransferase-10
VEGFR	vascular endothelial growth factor
WNT1	Wnt family member 1
